# An integrated fluid–structure interaction and thrombosis model for type B aortic dissection

**DOI:** 10.1007/s10237-021-01534-5

**Published:** 2022-01-25

**Authors:** Mei Yan Chong, Boram Gu, Chlöe Harriet Armour, Socrates Dokos, Zhi Chao Ong, Xiao Yun Xu, Einly Lim

**Affiliations:** 1 Department of Biomedical Engineering, University of Malaya, Kuala Lumpur, Malaysia; 2 Department of Chemical Engineering, Imperial College London, London, UK; 3 School of Chemical Engineering, Chonnam National University, Gwangju, Republic of Korea; 4 Graduate School of Biomedical Engineering, University of New South Wales, Sydney, Australia; 5 Department of Mechanical Engineering, University of Malaya, Kuala Lumpur, Malaysia

**Keywords:** Aortic dissection (AD), Fluid–structure interaction (FSI), Computational fluid dynamics (CFD), Intimal flap motion, Thrombus formation

## Abstract

False lumen thrombosis (FLT) in type *B* aortic dissection has been associated with the progression of dissection and treatment outcome. Existing computational models mostly assume rigid wall behavior which ignores the effect of flap motion on flow and thrombus formation within the FL. In this study, we have combined a fully coupled fluid–structure interaction (FSI) approach with a shear-driven thrombosis model described by a series of convection–diffusion reaction equations. The integrated FSI-thrombosis model has been applied to an idealized dissection geometry to investigate the interaction between vessel wall motion and growing thrombus. Our simulation results show that wall compliance and flap motion can influence the progression of FLT. The main difference between the rigid and FSI models is the continuous development of vortices near the tears caused by drastic flap motion up to 4.45 mm. Flap-induced high shear stress and shear rates around tears help to transport activated platelets further to the neighboring region, thus speeding up thrombus formation during the accelerated phase in the FSI models. Reducing flap mobility by increasing the Young’s modulus of the flap slows down the thrombus growth. Compared to the rigid model, the predicted thrombus volume is 25% larger using the FSI-thrombosis model with a relatively mobile flap. Furthermore, our FSI-thrombosis model can capture the gradual effect of thrombus growth on the flow field, leading to flow obstruction in the FL, increased blood viscosity and reduced flap motion. This model is a step closer toward simulating realistic thrombus growth in aortic dissection, by taking into account the effect of intimal flap and vessel wall motion.

## Introduction

Aortic dissection is a severe injury caused by a tear in the inner layer of the aortic wall, the intima. High blood pressure subsequently forces the tear to dissect within the medial layer, forming a false lumen (FL). Under normal physiological conditions, the hemostatic system initiates to form a blood clot, known as thrombus, at the site of vascular injury to prevent excessive blood loss. Hemostasis involves complex interactions between platelets, coagulation proteins, local hemodynamic conditions and the vascular wall, that result in multiple interlinked reactions causing vasoconstriction, platelet adhesion, activation, aggregation, the coagulation cascade, fibrin deposition, stabilization and dissolution (Periayah et al. 2017; Tomaiuolo et al. 2017; Xu et al. 2008).

Computational fluid dynamics (CFD) studies on aortic dissection have demonstrated altered pathological hemodynamics in the FL such as highly disturbed flow, long residence time and abnormally low wall shear stress (WSS) (Chen et al. 2013; Tse et al. 2011; Cheng et al. 2010), which have all been identified as potential markers for predicting thrombus development (Rayz et al. 2008, 2010). Rayz et al. (2008) found for intracranial aneurysms that regions with increased flow residence time and low shear stress correlated strongly with areas of thrombus deposition observed in follow-up magnetic resonance scans. Naim et al. (2018) and Li et al. (2020) suggested that increased blood flow in the FL could reduce flow recirculation and stagnation, thereby preventing FL thrombosis in dissection patients following thoracic endovascular aortic repair (TEVAR). The factors that influence the amount of blood flow in the FL were related to re-entry tears (location, number and distance between the tears) (Naim et al. 2018) and the degree of aortic arch angulation (Li et al. 2020). Dissection patients with fewer re-entry tears, longer distances between the tears and a smaller aortic arch angle were observed to have higher chances of FL thrombosis. Therefore, these CFD models are useful in predicting the regions which are likely to form thrombus for dissection patients.

The extent of FL thrombosis has been closely associated with patient prognosis in multiple clinical studies involving both medical and endovascular treatments, with complete thrombosis demonstrating beneficial effects on patients’ recovery, while partial thrombosis being associated with a higher mortality rate due to increased aortic expansion and eventual rupture (Tsai et al. 2007; Trimarchi et al. 2013). However, the complex cascade of thrombosis cannot be fully addressed with a CFD model alone. There is a need to couple fluid flow with the transport of chemical species, especially platelets, through a series of mathematical models, in order to address the growing interest in understanding the detailed mechanism of thrombus growth over time.

Numerous mathematical models have been developed to simulate the hemostasis process in pathological conditions (e.g., aortic dissection and aneurysm) using various approaches, corresponding to different spatial scales: continuum—macroscopic scale (Biasetti et al. 2012; Menichini et al. 2016, 2018; Menichini and Xu 2016; Bedekar et al. 2005), lattice Boltzmann—cellular scale (Ouared et al. 2008) and multiscale—a combination of macroscopic, cellular and subcellular scales (Zheng et al. 2020). Among these, the continuum approach is widely adopted due to its simplicity and efficiency in studying the macroscopic interaction of blood flow with biochemical species. The continuum models couple the Naiver-Stokes with convection–diffusion reaction equations, which are expressed in the form of partial differential equations, for fluid dynamics and species spatiotemporal concentration evolution, respectively.

Biasetti et al. (2012) presented a comprehensive model for thrombus formation in an idealized 2D-axisymmetrical abdominal aortic aneurysm (AAA) by modeling a total of 18 plasma-phase and surface-bound enzymes and zymogens that are involved in the coagulation cascade. They showed that the evolution of vortical structures convected thrombin through the domain and led to high concentrations in the distal portion of the AAA. This model was a comprehensive depiction of the coagulation cascade; however, this level of detail would demand excessive computational resources when applied to complex patient geometries together with realistic boundary conditions (Menichini et al. 2016, 2018; Bedekar et al. 2005). Bedekar et al. (2005) accomplished the framework of integrating a patient-specific cerebral aneurysms geometry within a relatively simple thrombotic cascade that was capable of modeling platelet activation, surface adhesion, thrombin generation and prothrombin inhibition. The application of such multi-physics methodologies to predict thrombus formation and growth patterns in the field of type *B* aortic dissection was first developed by Menichini and Xu (2016) in 2D phantom models with varying tear size and location, and further verified using medically managed (Menichini et al. 2016) and endovascularly treated (Menichini et al. 2018) patient-specific data. Their thrombosis model employed a reduced number of equations, while still being able to capture the FL clotting patterns in dissection patients within a clinically relevant timeframe (Menichini et al. 2016, 2018; Menichini and Xu 2016). The thrombus growth was regulated through a feedback mechanism, which allowed the key species, bound platelets (BP), to accumulate in regions of high concentration of activated platelets, low shear (e.g., local time-averaged wall shear stress (TAWSS) $<$ 0.2 Pa and bulk shear rate $<$ 50 s^−1^ (Menichini and Xu 2016)) and long residence time. All the aforementioned continuum models, however, neglect the compliant nature of the aortic wall (Biasetti et al. 2012; Menichini et al. 2016, 2018; Menichini and Xu 2016; Bedekar et al. 2005), which is expected to have a strong influence on the flow and pressure distributions in a dissected aorta (Alimohammadi et al. 2015; Chen et al. 2016; Qiao et al. 2019; Baumler et al. 2020; Bonfanti et al. 2019; Rudenick et al. 2015; Chong et al. 2020).

The common rigid wall assumption motivated the current continuum-based thrombus model to incorporate vessel wall motion using a fully coupled monolithic fluid–structure interaction (FSI) computational framework presented in our previous work (Chong et al. 2020). By applying the fully integrated FSI-thrombosis model to an idealized acute type *B* aortic dissection model, we aim to investigate the initiation and evolution of thrombus in the false lumen under the influence of detailed local hemodynamics and its dynamic interaction with a mobile flap and distensible vessel wall flap. Comparison between rigid wall and FSI simulations are performed in order to assess how the compliant wall behavior affects predictions of thrombus formation.

## Methodology

### Model geometry, fluid and wall models

**Fig. 1:**
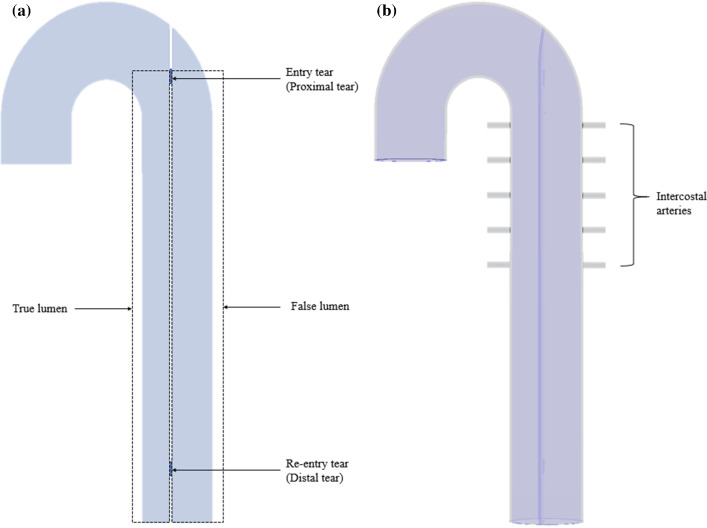
Idealized dissection model used in this study. **a** Fluid model; **b** Wall models—in *blue*, fluid–structure interface boundary; in gray, structure interface

A 3D idealized model of acute dissection was used in this study (Fig. 1), and a detailed description of the model can be found in (Chong et al. 2020). Briefly, the acute dissection model consisted of two parallel flow channels: a true lumen (TL, 19.4 mm diameter) and a FL (23 mm diameter), which had a common interface representing the intimal flap. The TL and FL were connected through two tears (10 mm diameter): an entry tear located proximally and a re-entry tear located distally along the intimal flap. Solid models were constructed by assuming a uniform wall thickness of 1.6 mm and a uniform flap thickness of 0.8 mm. Five pairs of intercostal arteries were included in the thoracic aorta to provide tissue tethering during the simulation.

### Thrombosis model

Thrombus formation was simulated using the shear-driven model developed by Menichini et al. (2016, 2018; Menichini and Xu 2016). While the original model simplified the overall thrombosis process through five transport species (residence time (RT), resting platelets (RP), activated platelets (AP), coagulant (*C*) and bound platelets (BP)), it is still computationally demanding, especially when integrated with a fully coupled FSI framework. Consequently, the current model has been further simplified to include four species (AP, RP, *C* and BP) in order to improve computational efficiency without affecting the thrombus growth pattern or final thrombus volume. Details about the impact of neglecting RT from the original model can be found in Supplementary Material.

The transport species in the flow field are described by the following convection–diffusion reaction (CDR) equation:

1 $$\frac{{\partial C_{i} }}{{{\text{d}}t}} + u_{{{\text{fluid}}}} \cdot \nabla C_{i} = D_{i} \nabla^{2} C_{i} + S_{i}$$

where $C_{i}$ is the concentration of species *i*,$u_{{{\text{fluid}}}}$ is the velocity field, $D_{i}$ is the diffusivity of species *i,* and $S_{i}$ is the reaction source term of species i.

The CDR equations for species C and BP are represented by Eqs. (2) and (3), respectively. C is diffusion-driven, whereas BP is an immobile species.

2 $$\frac{{\partial C_{i} }}{{{\text{d}}t}} = D_{i} \nabla^{2} C_{i} + S_{i}$$

3 $$\frac{{\partial C_{i} }}{{{\text{d}}t}} = S_{i}$$

The most CDR parameter values are available from the literature, and some have been modified by Menichini et al. (2016, 2018; Menichini and Xu 2016) to artificially accelerate the rate of thrombus growth. The CDR parameter values used in the current study are listed in Tables 1, 2 and 3, and changes made relative to Menichini’s original model have been validated against patient-specific rigid simulation results (Armour et al. 2020, 2021).

**Table 1:** Source terms for transport species

Species	Abbreviation	*S*_*i*_ form
Resting platelets	RP	$k_{{{\text{RP}}}} \left[ {{\text{AP}}} \right]\left[ {{\text{RP}}} \right]$
Activated platelets	AP	$k_{{{\text{AP}}}} \left[ {{\text{AP}}} \right]\left[ {{\text{RP}}} \right]$
Coagulant	*C*	$k_{C1} \emptyset_{C1} \left[ {{\text{AP}}} \right] + k_{C2} \emptyset_{C2} \left[ {{\text{AP}}} \right]\left( {1 - \emptyset_{{\dot{\gamma }}} } \right)$
Bound platelets	BP	$k_{{{\text{BP}}}} \emptyset_{{{\text{BP}}}} \emptyset_{{\dot{\gamma }}} \left[ {{\text{AP}}} \right]$

**Table 2:** Diffusion coefficient of species and kinetic constants

Species	*D*_*i*_ (m^2^ s^−1^)	Kinetic constant, *k*_*i*_		
		Symbol	Value	Units
RP	$1.6 \times 10^{ - 13}$	$k_{{{\text{RP}}}}$	− 0.15	s^−1^
AP	$1.6 \times 10^{ - 11}$	$k_{{{\text{AP}}}}$	3.0	s^−1^
*C*	$1.0 \times 10^{ - 8} \emptyset_{{\dot{\gamma }}}$	$k_{C1}$	16.0	nmol L^−1^ s^−1^
		$k_{C2}$	− 6.0	nmol L^−1^ s^−1^
BP	–	$k_{{{\text{BP}}}}$	12.0	nmol L^−1^ s^−1^

**Table 3:** Definition of symbols and parameter values

Symbol	Parameters	Value/expression	Units
$k_{{c_{wall} { }}}$	Flux in for coagulant	16.0	nmol L^−1^ m^−1^ s^−1^
$\dot{\gamma }_{t}$	Shear rate threshold	50.0	s^−1^
$AP_{t}$	AP threshold	15.0	
$C_{t}$	Coagulant threshold	10.0	nmol/L
$BP_{t}$	BP threshold	20.0	nmol/L
$\emptyset_{C}$	Switching coefficient for coagulant	$\emptyset_{C1} = \emptyset_{C} \left( {BP} \right)$ $\emptyset_{C2} = \emptyset_{C} \left( C \right)$	–
$\emptyset_{BP}$	Switching coefficient for BP	$\emptyset_{BP} = \emptyset_{BP} \left( {AP,C} \right)$	–
$\emptyset_{{\dot{\gamma }}}$	Switching coefficient for shear rate	$\emptyset_{{\dot{\gamma }}} = \frac{{\dot{\gamma }_{t}^{2} }}{{\dot{\gamma } + \dot{\gamma }_{t}^{2} }}$	–

Switching functions $(\emptyset_{i} )$ were introduced in the *C* and BP source terms to turn on and off the thrombus formation process gradually depending on the local environment.

4 $$\emptyset_{i} = \prod \frac{{C_{i}^{2} }}{{C_{i}^{2} + C_{{{\text{it}}}}^{2} }}$$

where $C_{{{\text{it}}}}$ is the defined threshold for respective transport species.

### Incorporating the effect of thrombus growth on flow

The Naiver-Stokes equation was adapted by introducing a negative fictitious force term $F$ to account for the effects of growing thrombus on blood flow as follows:

5 $$\rho_{{{\text{fluid}}}} \frac{{\partial u_{{{\text{fluid}}}} }}{\partial t} + \rho_{{{\text{fluid}}}} \left( {u_{{{\text{fluid}}}} \cdot \nabla } \right)u_{{{\text{fluid}}}} = \nabla .\sigma_{{{\text{fluid}} }} - F$$

6 $$F = k_{M} \frac{{{\text{BP}}^{2} }}{{{\text{BP}}^{2} + {\text{BP}}_{t}^{2} }}u$$

where *u* is velocity in m/s, $\rho_{{{\text{fluid}}}}$ is fluid density in kg/m^3^, $\mu$ is dynamic viscosity in kg/(m.s), *F* is the body force in (kg.m)/s^2^, $\sigma_{{\text{fluid }}}$ is stress tensor in kg/(m.s^2^), $\nabla$ is gradient operator, ${\text{BP}}$ is bound platelets (growing thrombus), ${\text{BP}}_{t}$ is threshold for BP and *k*_*M*_ is a constant and is equal to 10^7^ kg/(m^3^.s). The subscript *fluid* denotes the variables and properties in the fluid domain.

### Computational details

Blood was treated as an incompressible non-Newtonian fluid described by the Quemada viscosity model with parameters taken from Neofytou (2004) and a density of 1060 kg/m^3^. Time-dependent flow waveform with a flat velocity profile was imposed at the model inlet (Chong et al. 2020). The waveform had a frequency of 1.02 Hz, Womersley number based on the inlet diameter of 26, and the corresponding Reynolds number *Re* had a maximum of 2694, with a mean of 448. The blood flow was considered laminar since *Re* was below the threshold for turbulent flow (Nerem et al. 2006). A TL outlet pressure waveform was calculated through the three-element Windkessel model: *C* (compliance), *R*_*P*_ (peripheral resistance) and *R*_*C*_ (characteristic impedance), following the same parameters in (Chong et al. 2020), while the FL outlet acted as a close-end wall. The transport species, AP and RP were initialized with a relative concentration of one and zero initial concentration was set for *C* and BP. Species were modeled at the wall by either specified concentration or surface flux boundary condition, which are listed in Table 4.

**Table 4:** Species boundary conditions

Species	Description
RP	A relative RP concentration of 2 was applied at the wall
AP	A relative AP concentration of 2 was applied at the wall
*C*	$$D_{{c_{{{\text{eff}}}} }} \left. {\frac{\partial C}{{\partial n}}} \right|_{{{\text{wall}}}} = \left\{ {\begin{array}{*{20}c} {k_{{c_{{{\text{wall}}}} }} } & {{\text{if}}\;{\text{TAWSS}} < 0.15\;P{\text{a}}\;{\text{and}}\;{\text{BP}} < {\text{BP}}_{t} } \\ 0 & {{\text{otherwise}}} \\ \end{array} } \right.$$ Flux boundary condition, with the flux depending on local TAWSS values of the previous cycle and the local concentration of BP
BP	Zero flux boundary condition at the wall

The aortic wall and intimal flap were assumed to behave like a linearly elastic isotropic material with a Young’s modulus (*E*) of 2.7 and 6.75 MPa, respectively (Chong et al. 2020). Tissue tethering of the aorta was taken into account by fixing the two ends and branches of intercostal arteries. To investigate the effect of flap mobility on thrombosis results, *E*_flap_ was increased to 60 MPa to simulate a less mobile flap.

For each model, an FSI simulation was performed first without including the thrombosis model. This required 15 cardiac cycles to obtain a periodic solution. The thrombosis model was then included, and fully coupled FSI-thrombosis simulations were run for at least 15 cardiac cycles until there was no further thrombus growth. Since mesh sensitivity tests for coupled FSI simulations of the same geometric model were reported in our previous work (Chong et al. 2020), the new mesh sensitivity test was focused on ensuring mesh independence for the predicted thrombus volume, which was the final product of the modeled chemical reactions. Details of the mesh sensitivity test on the rigid wall-thrombosis model can be found in Supplementary Material. The final adopted mesh contained 178 k tetrahedral elements and 8 prismatic layers near the wall.

The FSI and thrombosis models described above were implemented in COMSOL Multi-physics (v5.2, COMSOL AB, Sweden) which was used to solve all the relevant equations. Due to the complexity of modeling thrombus growth over time while accounting for wall motion, the multi-physics FSI-thrombosis model required approximately two to three months of computational time using the workstation Intel Xeon CPU E5-2665 @ 2.40 GHz, 96 GB RAM. Detailed comparisons between FSI (*E*_flap_ = 6.75 MPa) and rigid models were made, and the effect of flap mobility was then investigated by analyzing results from an additional FSI model (*E*_flap_ = 60 MPa). Here, we only present results obtained with the integrated FSI-thrombosis simulations.

## Results

### Flap displacement

**Fig. 2:**
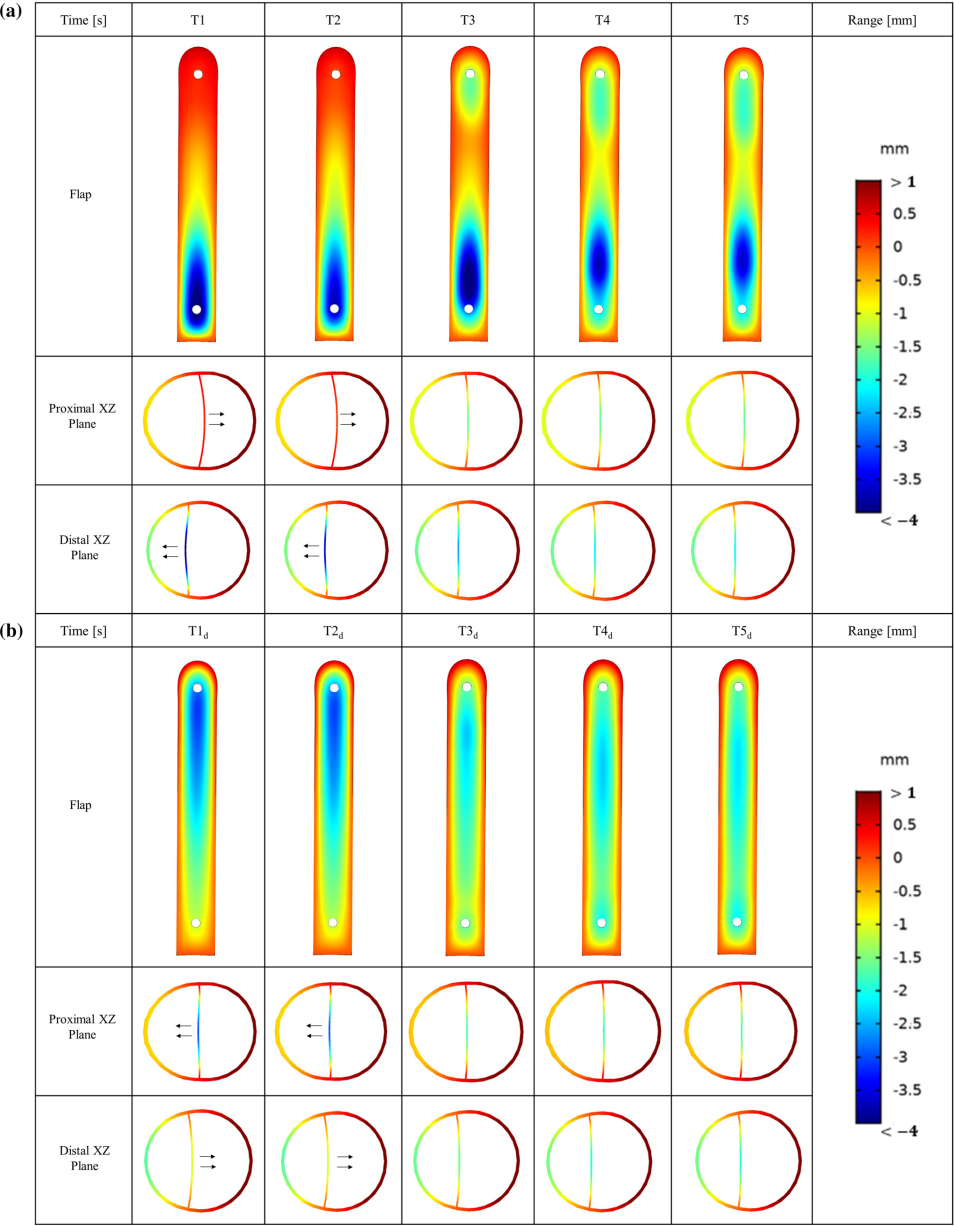
Flap displacement and configurations in the proximal and distal locations obtained from FSI model (*E*_flap_ = 6.75 MPa). The flap moves toward the TL (i.e., curved to the left, as indicated by the arrows) or vice versa. The selected time points, where **a** *T*1 = 2.130 s, *T*2 = 5.085 s, *T*3 = 8.040 s, *T*4 = 10.995 s and *T*5 = 13.950 s, corresponding to peak systole from 3rd to 15th cycle, with an interval of three cycles; **b** *T*1_*d*_ = 2.380 s, *T*2_*d*_ = 5.335 s, *T*3_*d*_ = 8.290 s, *T*4_*d*_ = 11.285 s and *T*5_*d*_ = 14.240 s, corresponding to early diastole from 3rd to 15th cycle, with an interval of three cycles

**Fig. 3:**
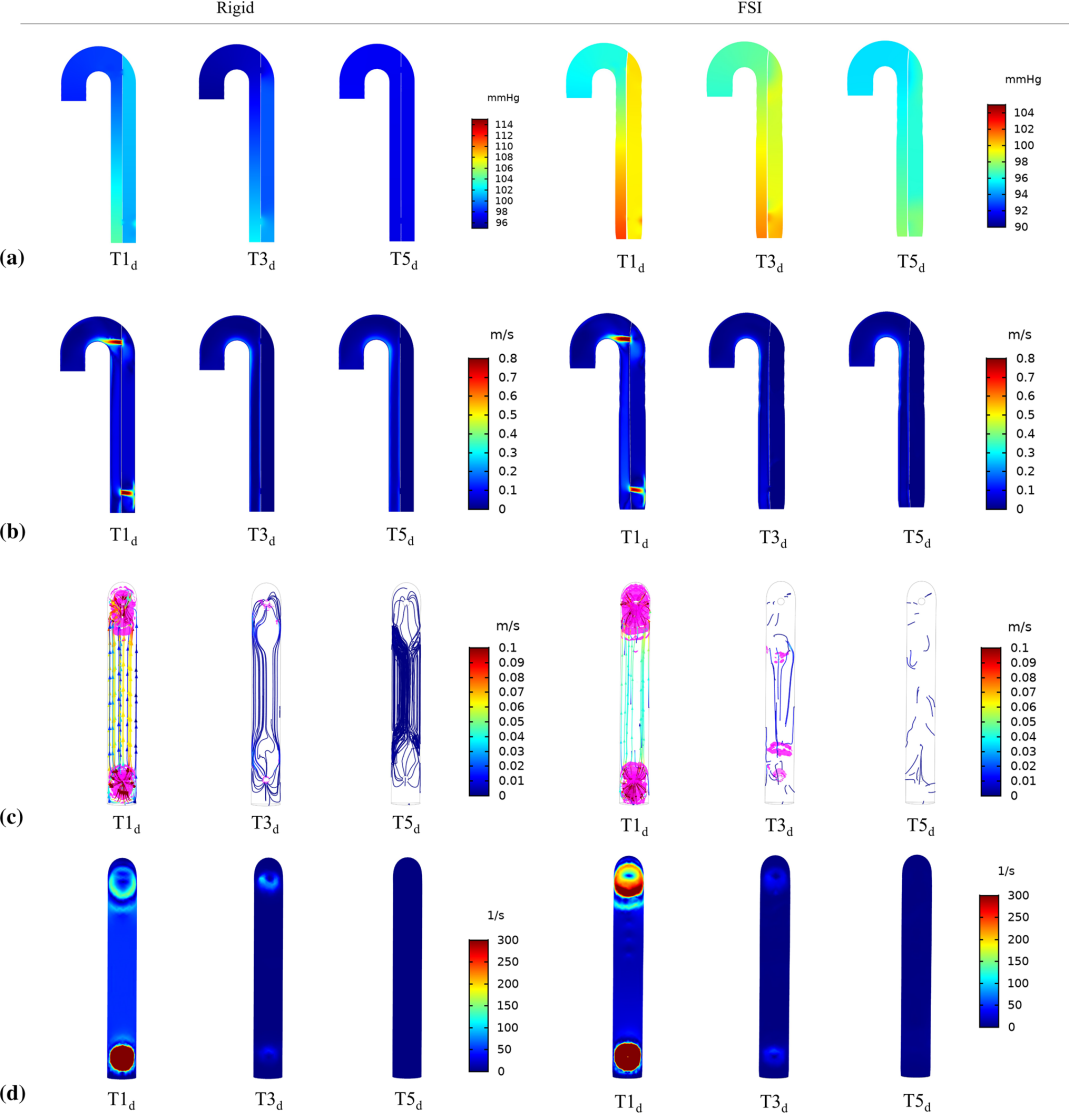
The evolution of **a** pressure, **b** velocity, **c** vortex formation *(highlighted in magenta)* superimposed on instantaneous streamlines and **d** vorticity, predicted by rigid and FSI (*E*_flap_ = 6.75 MPa) models, respectively. The selected time points, where *T*1 = 2.130 s, *T*3 = 8.040 s and *T*5 = 13.950 s, corresponding to peak systole from the 3rd to 15th cycle, with an interval of six cycles

**Fig. 4:**
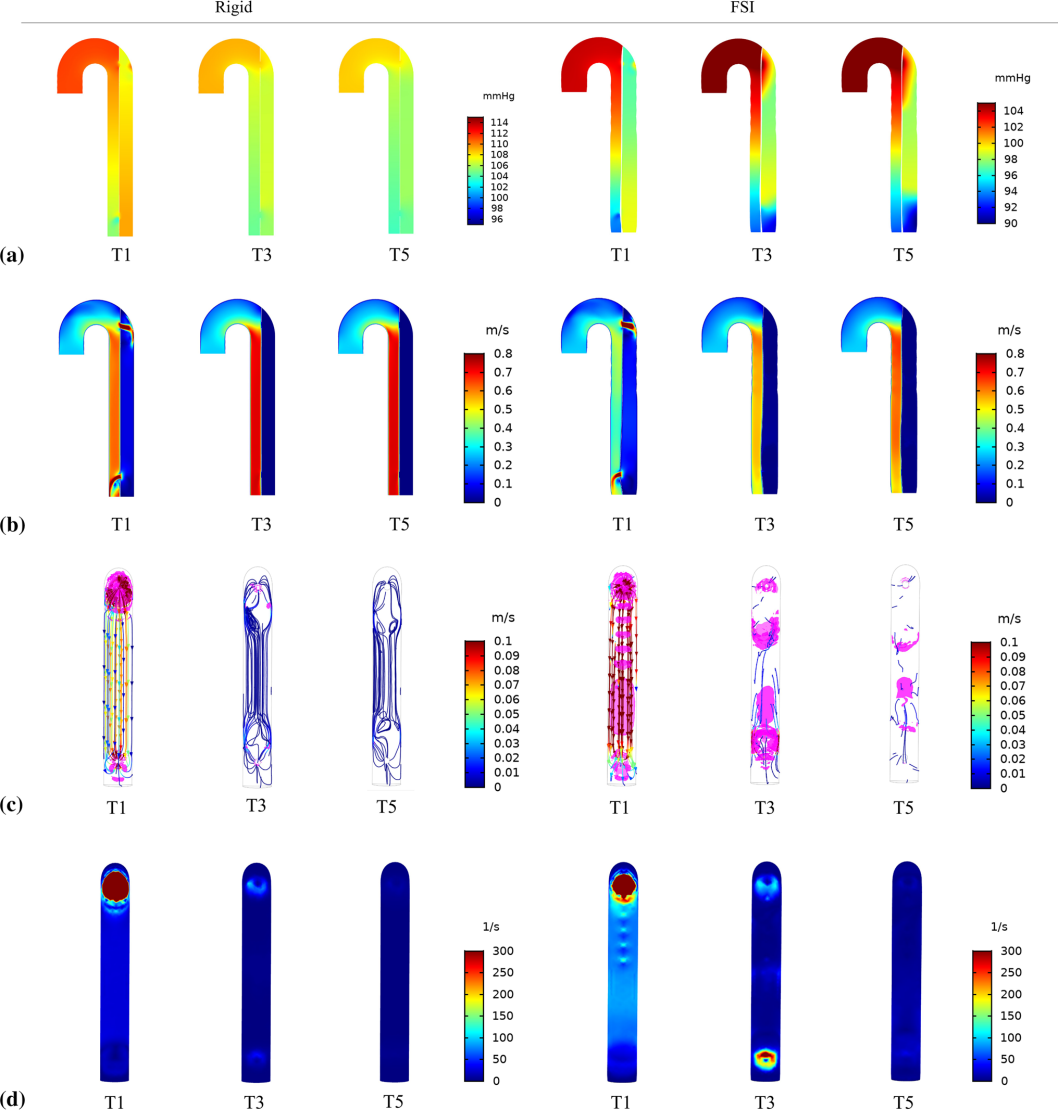
The evolution of **a** pressure, **b** velocity, **c** vortex formation *(highlighted in magenta)* superimposed on instantaneous streamlines and **d** vorticity, predicted by rigid and FSI (*E*_flap_ = 6.75 MPa) models, respectively. The selected time points, where *T*1_*d*_ = 2.380 s, *T*3_*d*_ = 8.290 s and *T*5_*d*_ = 14.240 s, corresponding to early diastole from the 3rd to 15th cycle, with an interval of six cycles

**Fig. 5:**
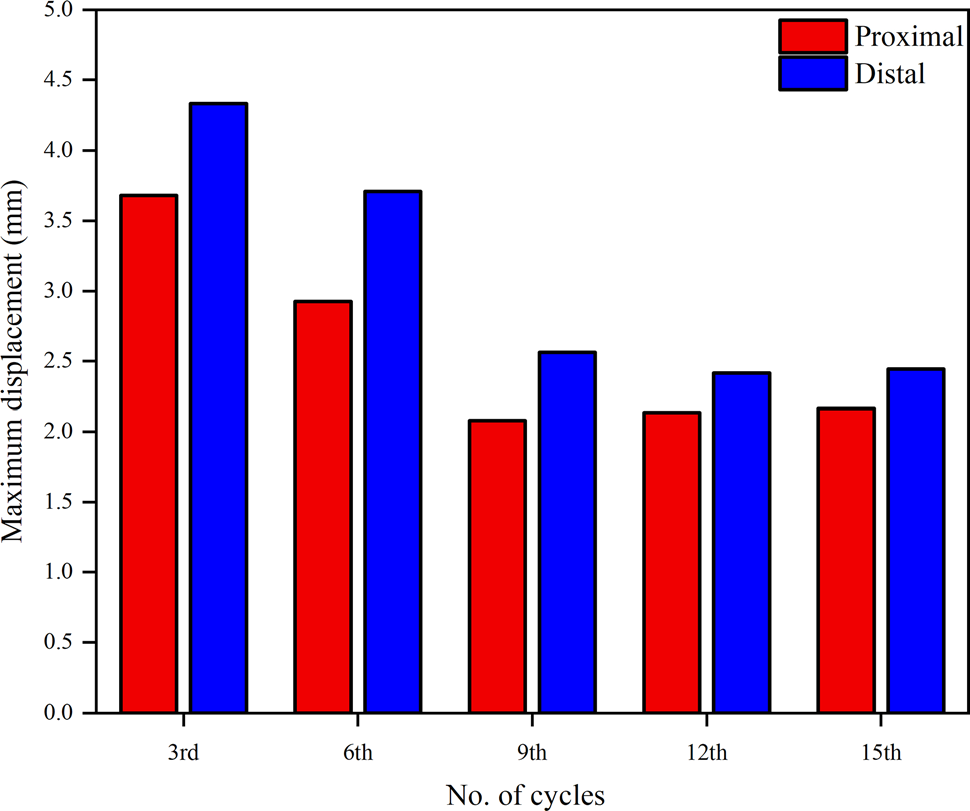
Maximum displacement at the proximal and distal cut-plane locations from the 3rd to 15th cycle, with an interval of three cycles (*E*_flap_ = 6.75 MPa)

Figure 2 shows the flap configurations at peak systole and early diastole at time points *T*1-*T*5, which range from the 3rd to the 15th cycle. Flap motion was strongly affected by the pressure difference between TL and FL (Figs. 3a and 4a). At peak systole, greater pressure in the proximal TL caused the flap to be pushed toward the FL, while FL pressure predominantly exceeded the TL in the distal location, hence the flap moved toward the TL. Flow reversal occurred at early diastole, resulting in reversed flap configuration within the same cycle. The periodic flap motion gradually diminished as shown in Fig. 5, once thrombus started to grow in the FL from *T*1. In addition, after *T*4 the location of maximum flap displacement shifted from the distal tear toward the middle FL region where no thrombus was formed.

### Flow and pressure

The evolution of pressure, velocity, vortex formation and vorticity was substantially different between the rigid and FSI models as shown in Figs. 3, 4. The most obvious difference was the continuous presence of vortex in the FSI model from the 3rd to 15th cycle, which was identified using $\lambda_{2}$ criterion (Jeong and Hussain 2006; Cucitore et al. 1999) with a threshold of − 10 s^−2^. Flap motion caused the vortices to spread along the descending aorta at *T*1, resulting in lower vorticity (maximum vorticity = 2740.44 s^−2^) nearby the proximal tear when compared to the rigid model (maximum vorticity = 3109.99 s^−2^). During *T*1_*d*_, the maximum vorticity was located nearby the distal tear, the magnitude of which was higher in the rigid model (1843.76 s^−2^ rigid vs. 1656.78 s^−2^ FSI). The vortices traveled downstream and decayed from *T*3 to *T*5, with the FSI model having much higher vorticity magnitude.

**Fig. 6:**
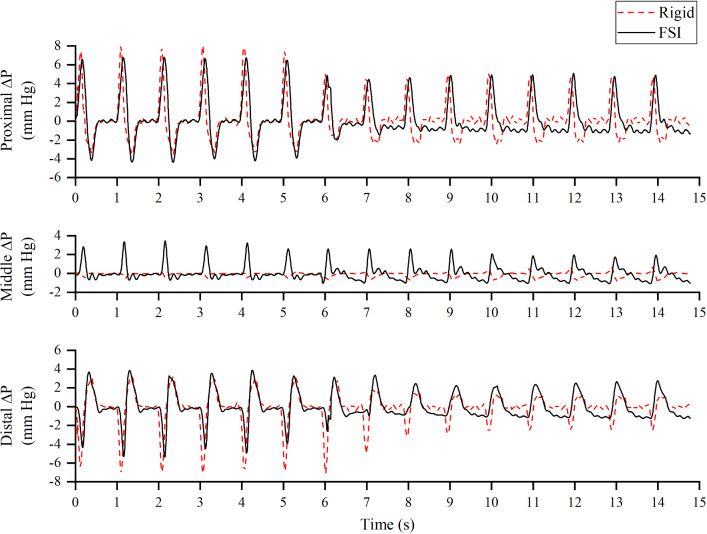
Spatial-averaged pressure difference between true lumen and false lumen, $\Delta P = P_{{{\text{TL}}}} - P_{{{\text{FL}}}}$, evaluated at proximal, middle and distal locations for both rigid and FSI (*E*_flap_ = 6.75 MPa) models

In addition, the velocity jet around the tears disappeared after *T*1_*d*_ when there was significantly less flow entering the FL through proximal tear. The spatial-averaged pressure difference between TL and FL ($\Delta P = P_{{{\text{TL}}}} - P_{{{\text{FL}}}}$) was evaluated at three locations: proximal (at the level of the proximal tear), middle (85.5 mm below the inlet surface) and distal (at the level of the distal tear) and the results are shown in Fig. 6. A general trend was the reduction in pressure difference after 6 s in all locations. Comparison of pressure difference between the rigid and FSI models revealed increased deviation from proximal to distal location—notably the flap motion was the greatest at the distal location.

### Time-averaged activated platelets, shear rates and wall shear stress

**Fig. 7:**
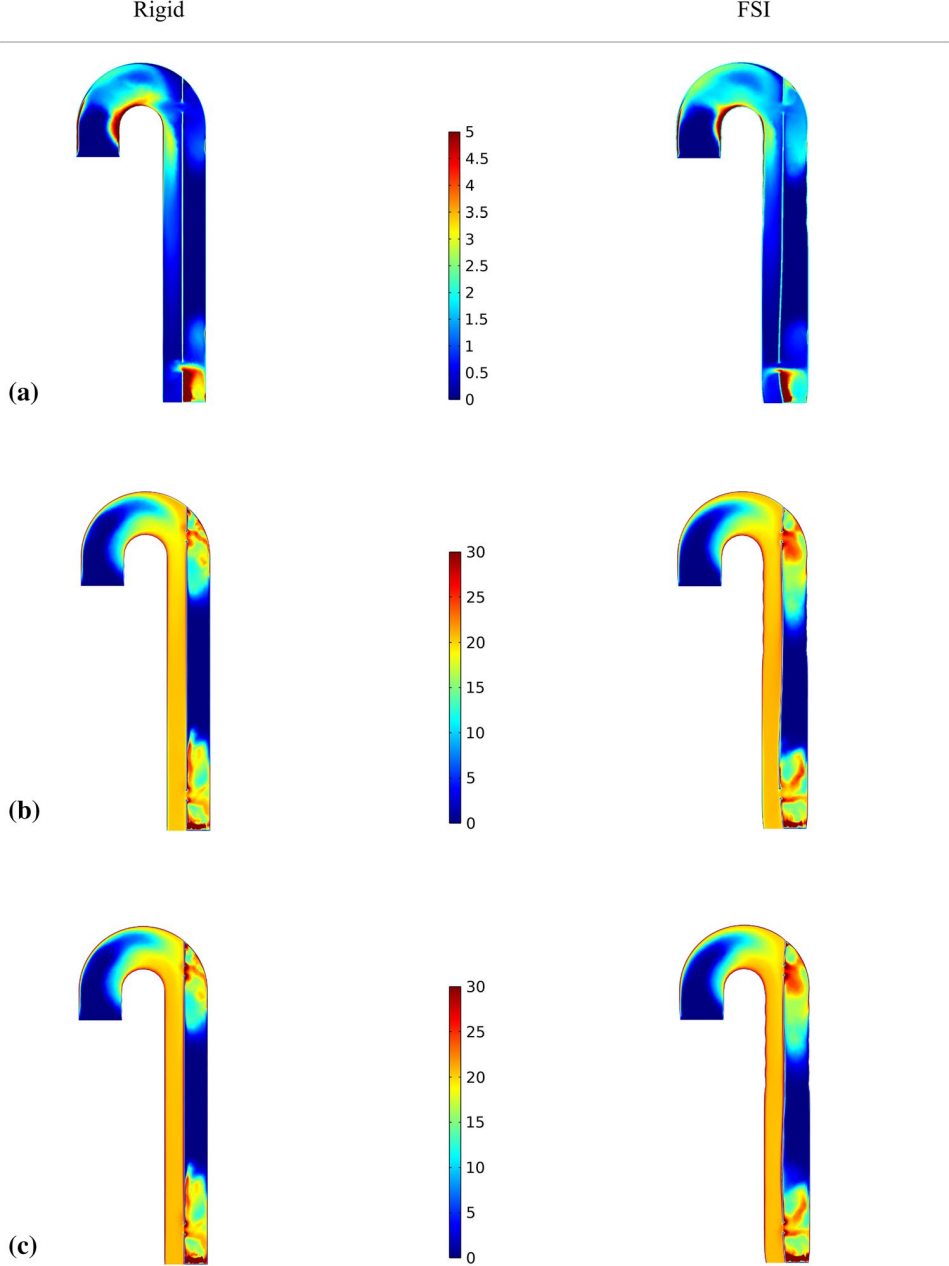
Cycle-averaged distribution of activated platelets at the **a** 3rd, **b** 9th and **c** 15th cycle, for rigid and FSI (*E*_flap_ = 6.75 MPa) models, respectively

**Fig. 8:**
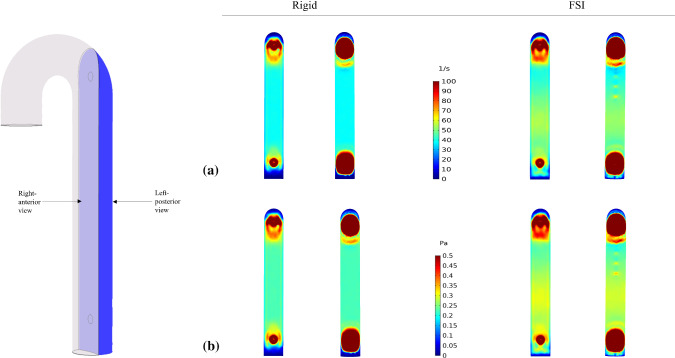
Cycle-averaged distribution of **a** shear rates and **b** wall shear stress (WSS), for rigid and FSI (*E*_flap_ = 6.75 MPa) models, respectively. The shear rates and WSS distributions contain a right-anterior view (*left*) and left-posterior view (*right*) of the false lumen

High velocity gradient in proximity to the tears caused APs to be transported and accumulate in regions of low shear rate and WSS, located at both ends of the FL at the 3rd cycle (Fig. 7a). More APs were transported over time (Fig. 7b, c), except in the middle section of FL, where the driving force may not be sufficient to convect APs from its initial source into the middle FL section. Figure 8 shows cycle-averaged shear rates and shear stress distributions in the FL. As expected, the highest shear values were observed when jet flow impinged upon the FL wall opposite to the proximal tear. The flap motion in the FSI model increased the shear values by up to 17% in the FL, especially near the proximal tear. These high shear values caused APs to distribute more uniformly and further away from the proximal location.

### Thrombus growth over time

**Fig. 9:**
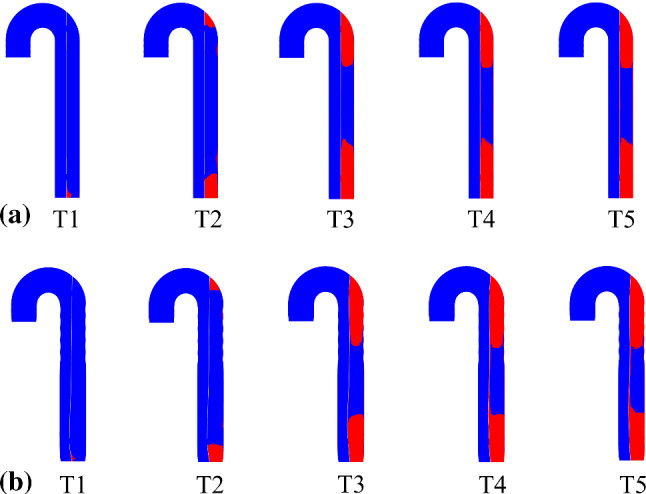
Thrombus growth over time for **a** rigid and **b** FSI (*E*_flap_ = 6.75 MPa) models respectively, at *T*1 = 2.130 s, *T*2 = 5.085 s, *T*3 = 8.040 s, *T*4 = 10.995 s and *T*5 = 13.950 s. In *red*, areas where thrombus forms; in *blue*, areas where blood flows

**Fig. 10:**
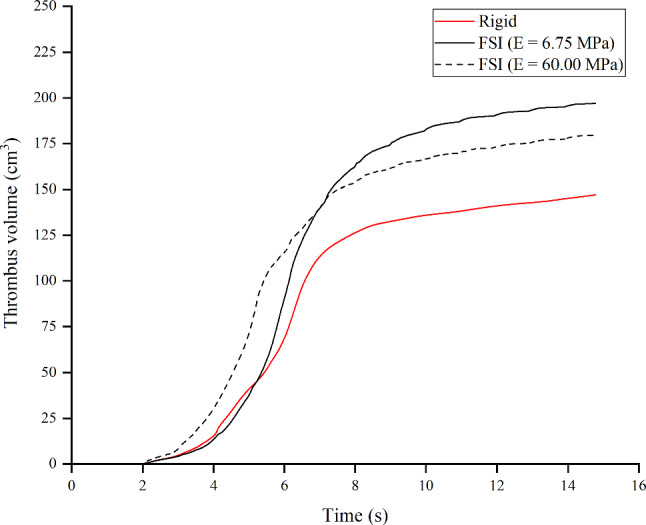
Temporal variation of thrombus volume for rigid and FSI models

Thrombus started to grow at both ends of the FL due to high AP concentration along with low shear rates and TAWSS, and slowly expanded toward the middle FL as shown in Fig. 9. Partial thrombosis was achieved in both FSI and rigid models with different thrombus growth rates. Figure 10 shows the thrombus volume over time for each model. From this, two different growth rates can be distinguished clearly: from *T*1 to *T*2, the rigid model predicted slightly faster thrombus growth than FSI models; after *T*2, the rigid model became significantly slower. The exact time point for the flexible FSI model to overtake the rigid model was 5.225 s. An additional FSI model with a stiffer flap (*E*_flap_ = 60 MPa) was simulated to investigate the effect of flap mobility on thrombus formation. The thrombus volume from this additional FSI simulation is also shown in Fig. 10. It can be seen that during the first 7.155 s of simulation time, the increased flap stiffness caused faster thrombus formation. However, after 7.155 s the growth rate slowed compared to the model with a more mobile flap.

**Fig. 11:**
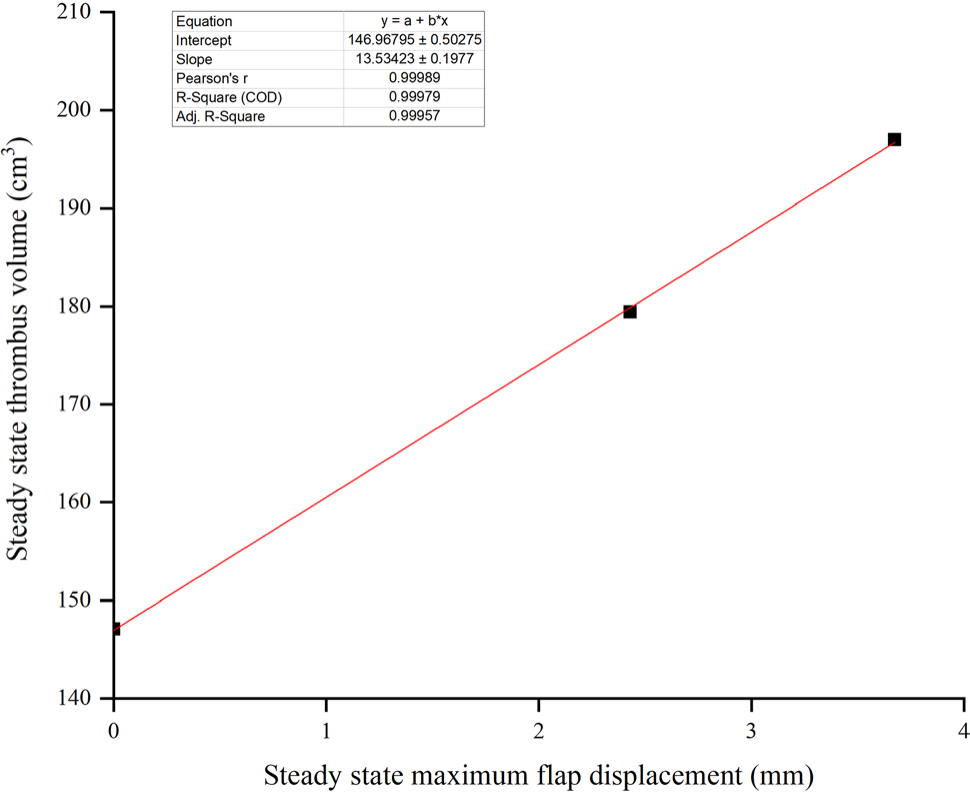
Effect of maximum flap displacement on the thrombus volume. The data is taken at the 15th cycle when steady state is reached. The linear fit line equation is *y* = 146.97 + 13.53 × with *R*^2^ = 0.9998

The thrombus almost stopped growing after the 15th cycle, where the thrombus volume predicted by the FSI models with Young’s modulus *E*_flap_ of 6.75 MPa and 60 MPa were 25.3% and 18.0% greater than the rigid model, respectively. The relationship between thrombus volume and maximum flap displacement is illustrated in Fig. 11. Thrombus volume was evaluated after a steady state was achieved for all models with a maximum flap displacement of 0 (rigid model), 2.43 mm (*E*_flap_ = 60 MPa) and 3.67 mm (*E*_flap_ = 6.75 MPa). Thrombus volume and maximum flap displacement were linearly related and can be represented by the equation of *y* = 146.97 + 13.53*x*, where *y* = thrombus volume (cm^3^) and *x* = maximum flap displacement (mm). The coefficient of determination for the resulting relationship was adequate with *R*^2^ = 0.9998.

### Effect of thrombus growth on blood viscosity

**Fig. 12:**
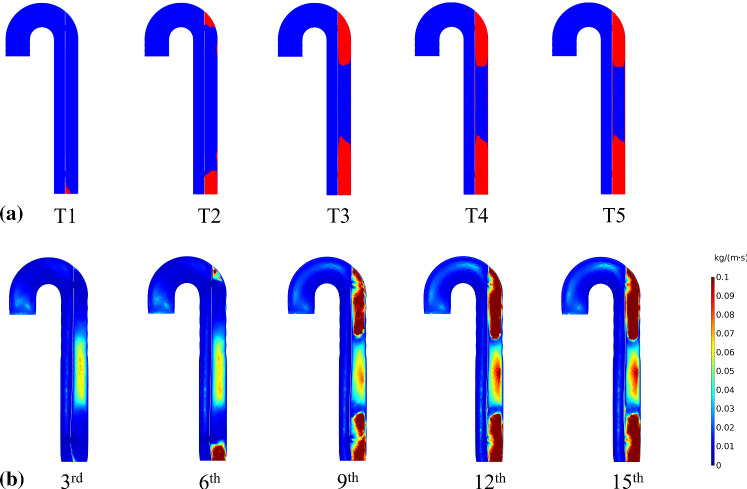
Relationship between **a** thrombus growth over time for FSI (*E*_flap_ = 6.75 MPa) model and **b** cycle-averaged blood viscosity. For **a**, in *red*, areas where thrombus forms; in *blue*, areas where blood flows, at *T*1 = 2.130 s, *T*2 = 5.085 s, *T*3 = 8.040 s, *T*4 = 10.995 s and *T*5 = 13.950 s. For **b**, the distribution plot is visualized from 3rd to 15th, at an interval of three cycles

The formation and growth of thrombus in the FL altered its geometry and local shear rate, thereby affecting blood viscosity. Changes in viscosity in response to thrombus growth can be seen in Fig. 12. Initially, viscosity was low in the vicinity of proximal and distal tears due to high shear rates when blood entered and left the FL as a high velocity jet. As the thrombus grew in size, both ends of the FL thrombosis, leading to increased viscosity in these regions. Thrombus growth resulted in notable change in blood viscosity within the FL, but the TL was hardly affected.

## Discussion

In this study, we present a challenging multi-physics model that, for the first time, combines fully coupled FSI with the transport of chemical species to simulate the interaction of drastic flap motion with growing thrombus in the acute dissection scenario. By implementing a simplified thrombus model in our previously reported FSI work (Chong et al. 2020), we aimed to elucidate the role of intimal flap motion in FL thrombosis. Thrombus initiation and growth within the FL are modeled through platelet activation and deposition depending on the cycle-averaged WSS and shear rates, as well as the distribution of activated and resting platelets.

The drastic flap motion and aortic wall expansion/contraction (Fig. 2) affected distributions of pressure, velocity, vortex formation, cycle-averaged APs, shear rates and WSS (Figs. 3, 4, 7 and 8). Large spatial variations in shear rate and shear stress were crucial in thrombus formation; high values triggered platelet activation, while very low values allowed accumulation of APs, and hence thrombus growth. Platelets exposed to high shear values in proximity to the tears became activated and later trapped in flow recirculation regions, which were initially located at both ends of the FL.

Thrombus gradually expanded toward the middle section of the FL and kept growing in volume, resulting in less flow entering the FL. We observed that flap displacement was significantly reduced at the proximal and distal cut-plane locations once thrombus occupied more than 50% of the FL volume (Fig. 5). This is because the cross-lumen pressure difference in all locations decreased (Fig. 6). The reduced flap displacement still caused the continuous presence of vortices until 14.2 s, resulting in obvious differences in thrombus growth rate between the rigid and FSI models within the time period of 5.225–14.775 s (Figs. 9 and 10). The continuous presence of vortices in the FSI model meant that APs could be transported further away from the proximal FL into the middle section. Conversely, the rigid model lacked the momentum to convect APs through the domain once the vortices had dissipated, causing thrombus growth to slow down. An interesting difference between rigid and FSI models in terms of pressure difference was the increasing deviation from proximal to distal location. The greater deviation in the distal location was highly affected by the drastic flap motion which caused TL compression at peak systole. This reiterates the conclusion that vessel wall motion should not be ignored in the hemodynamic analysis of acute dissection patients, as we reported in our previous work (Chong et al. 2020).

The evolution of thrombus in this acute dissection model went through three distinct phases (Fig. 10): time-lag, accelerated and plateau, which is consistent with the finding reported by Biasetti et al. (2012). Flap motion up to 4.45 mm caused increased thrombus growth during accelerated phase, which resulted in a relatively higher plateau value of 196.97 cm^3^ compared to the rigid model of 147.11 cm^3^. Since the Young’s modulus of the flap is expected to affect the thrombus growth, a parametric study of *E*_flap_ was conducted to simulate three different scenarios: rigid flap, stiffer flap (*E*_flap_ = 60 MPa) and flexible flap (*E*_flap_ = 6.75 MPa) (Figs. 10 and 11). Our linear regression analysis suggested a positive linear relationship between thrombus volume and the maximum flap displacement. This particular relationship may not hold for more complex geometry when anatomic features of individual patients are taken into account, such as curvature of the vessel and the number of re-entry tears, which are likely to influence vortex formation, and hence thrombus growth.

Another interesting observation was that a stiffer flap resulted in faster thrombus growth before 7.155 s. The slower initial growth rate with the compliant flap (*E*_flap_ = 6.75 MPa) maybe due to the drastic flap motion creating a strong high velocity jet entering the FL through the proximal entry tear, thus reducing residence time inside the FL. As discussed previously, thrombus formation requires a combination of high AP concentration, low WSS and high residence time. As the thrombus grew over time, the high velocity jet dampened and the flap motion reduced progressively. The displacement of the compliant flap was no longer high enough that it hindered thrombus formation, instead it created more disturbed flow which provoked persistent vortex development that favored platelet activation and thrombus growth, leading to an overall larger volume of thrombus formation compared to the stiffer flap.

The effect of thrombus growth on blood viscosity is seldom quantified so far. In the current work, the Quemada non-Newtonian viscosity model with 45% hematocrit (HCT) was employed to describe the shear-thinning behavior of blood. Gradual progression of FL thrombosis caused an overall reduction in shear rate. The tendency of erythrocytes to aggregate at low shear rates contributed to higher blood viscosity within the FL, with an average value of 0.0199 kg/(m.s) (Fig. 12). Blood viscosity in the TL was not affected by thrombus formation in the FL and remained fairly constant at 0.0049 kg/(m.s). Note that the average blood viscosity values found here corresponded to a representative HCT of 45%. The choice of HCT value, ranging between 30 and 55%, is likely to have an influence on thrombus formation (Jafarinia et al. 2020).

Despite differences in thrombus growth rate, all models predicted partial thrombosis with the middle section of FL remaining patent. The flow is relatively organized in this region, preventing any vortex development which tends to favor thrombus formation. Similar initial thrombus growth patterns have been reported by Menichini et al. (2016) in their 2D idealized models with two tears, but our model predicted extended thrombus formation toward the middle section of FL. The possible reasons could be different inlet velocity waveforms and geometrical differences between the 2D and 3D models used in this study. Menichini et al. (2016) subsequently employed an acute dissection patient geometry with two tears (one proximal tear and one re-entry tear at the middle section) in a longitudinal three years follow-up study. Slight discrepancies between computational results and follow-up CT scans were observed near the proximal FL region, where neglecting the flap motion resulted in under-prediction of thrombus growth. In our FSI model (*E*_flap_ = 6.75 MPa), flap motion influences the distribution of hemodynamic parameters, especially near the proximal FL from 5.225 s onward. The persistent appearance of vortices caused by flap motion resulted in an increase in thrombus volume by 25.3% compared to the rigid model. It is reasonable to expect that the FSI-thrombosis model will further improve the agreement between computational model predictions and in vivo observations. Several computational studies have highlighted wall compliance as an important factor for predictions of flow and thrombus growth in aortic aneurysms and dissections (Naim et al. 2018; Biasetti et al. 2012; Menichini et al. 2016, 2018; Menichini and Xu 2016; Armour et al. 2020; Wang et al. 2021).

The advancement in imaging modalities such as ECG-gated thoracoabdominal aorta CT angiography (CTA) (Yang et al. 2014; Ganten et al. 2009) and intravascular ultrasound (IVUS) (Lortz et al. 2019) are helpful in assessing wall compliance and intimal flap mobility. A recent study based on IVUS data showed that chronic type *B* dissection patients who presented with a highly mobile flap (amplitude of > 3 mm) prior to TEVAR had improved aortic remodeling and lower re-intervention rate (Lortz et al. 2019). This supported our computational results as increased flap motion promotes thrombus growth dynamics. The amount of flap motion might be of interest to stratify dissection patients into different risk categories. Hence, future studies will apply the current coupling approach in patient-specific cases to further validate and refine the model. The key point of our future studies is to advance the model applicability in the clinical setting by finding an optimal balance between computational efficiency and model accuracy. Further work is being conducted to assess the dependency of thrombus prediction on each of the modeled species to see if further simplification can be made without compromising model accuracy (Armour et al. 2021).

## Conclusion

This integrated FSI-thrombosis study represents the first attempt to predict thrombus formation and growth over time influenced by intimal flap motion. Flap motion affected flow and pressure distributions, and hence thrombus growth for aortic dissection. The flap-induced higher shear rates and shear stress around the tears caused more activated platelets to travel downstream. This greatly sped up thrombus growth in the FSI models during the accelerated phase compared to the rigid model. By varying the Young’s modulus of flap to simulate a rigid, stiffer and flexible flap, a linear relationship between thrombus growth and maximum flap displacement was found. A best fit linear equation was obtained with R^2^ close to 1.
